# Characterization of genome-wide phylogenetic conflict uncovers evolutionary modes of carnivorous fungi

**DOI:** 10.1128/mbio.02133-24

**Published:** 2024-08-29

**Authors:** Weiwei Zhang, Yani Fan, Wei Deng, Yue Chen, Shunxian Wang, Seogchan Kang, Jacob Lucas Steenwyk, Meichun Xiang, Xingzhong Liu

**Affiliations:** 1 State Key Laboratory of Medicinal Chemical Biology, Key Laboratory of Molecular Microbiology and Technology, and Department of Microbiology, College of Life Science, Nankai University, Tianjin, China; 2 State Key Laboratory of Mycology, Institute of Microbiology, Chinese Academy of Sciences, Beijing, China; 3 University of Chinese Academy of Sciences, Beijing, China; 4 Department of Plant Pathology & Environmental Microbiology, The Pennsylvania State University, University Park, Pennsylvania, USA; 5 Howards Hughes Medical Institute and Department of Molecular and Cell Biology, University of California, Berkeley, California, USA; Duke University, Durham, North Carolina, USA

**Keywords:** incomplete lineage sorting, horizontal gene transfer, nematode-trapping fungi

## Abstract

Mass extinction has often paved the way for rapid evolutionary radiation, resulting in the emergence of diverse taxa within specific lineages. The emergence and diversification of carnivorous nematode-trapping fungi (NTF) in Ascomycota have been linked to the Permian-Triassic (PT) extinction, but the processes underlying NTF radiation remain unclear. We conducted phylogenomic analyses using 23 genomes that represent three NTF lineages, each employing distinct nematode traps—mechanical traps (*Drechslerella* spp.), three-dimensional (3D) adhesive traps (*Arthrobotrys* spp.), and two-dimensional (2D) adhesive traps (*Dactylellina* spp.), and the genome of one non-NTF species as the outgroup. These analyses revealed multiple mechanisms that likely contributed to the tempo of the NTF evolution and rapid radiation. The species tree of NTFs based on 2,944 single-copy orthologous genes suggested that *Drechslerella* emerged earlier than *Arthrobotrys* and *Dactylellina*. Extensive genome-wide phylogenetic discordance was observed, mainly due to incomplete lineage sorting (ILS) between lineages. Two modes of non-vertical evolution (introgression and horizontal gene transfer) also contributed to phylogenetic discordance. The ILS genes that are associated with hyphal growth and trap morphogenesis (e.g., those associated with the cell membrane system and polarized cell division) exhibited signs of positive selection.

**IMPORTANCE:**

By conducting a comprehensive phylogenomic analysis of 23 genomes across three NTF lineages, the research reveals how diverse evolutionary mechanisms, including ILS and non-vertical evolution (introgression and horizontal gene transfer), contribute to the swift diversification of NTFs. These findings highlight the complex evolutionary dynamics that drive the rapid radiation of NTFs, providing valuable insights into the processes underlying their diversity and adaptation.

## INTRODUCTION

Mass extinctions result in vacated ecological niches that can be occupied by novel species and drive subsequent radiation events (1, 2). Mass extinction and concomitant radiations have been documented in multiple lineages, including angiosperms (3), planktic foraminifera (4), snakes (5), modern birds (6), and mushrooms (7). Comparative genomics has facilitated the systematic identification of candidate genetic changes underlying speciation and adaptive radiation (8).

Carnivorous nematode-trapping fungi (NTF) emerged after the Permian-Triassic (PT) extinction (ca. 252 million years ago) and radiated into multiple lineages that form distinct trapping devices to capture free-living nematodes (9). The emergence of NTF from saprophytic fungal species is thought to be driven by nematode proliferation followed by the PT extinction, an event that resulted in a carbon-rich and nitrogen-poor environment (10–13). The ability to capture and consume nematodes allows NTF to obtain extra nitrogen, likely conferring a competitive advantage over saprophytic fungi (9) and driving diversification. NTF have radiated into three clades that form distinct trapping systems: *Arthrobotrys* spp. that employ three-dimensional (3D) adhesive traps (networks), *Dactylellina* spp. that utilize two-dimensional (2D) adhesive traps (knob, column, non-constricting ring), and *Drechslerella* spp. that form constricting rings, mechanical traps (14).

Phylogenomics has greatly advanced our understanding of the Tree of Life, mechanisms of gene and genome evolution, and the relationship between genomic and phenotypic divergence during speciation (15, 16). Phylogenomics has also revealed why some genes exhibit evolutionary patterns distinct from the phylogenetic history of species carrying these genes (17, 18). Theoretical and empirical studies have shown that this discordance or incongruence can be caused by analytical errors (e.g., insufficient taxon sampling and gene tree estimation) and diverse biological factors. Among biological factors include incomplete lineage sorting (ILS), which causes ancestral genetic variations to persist during rapid speciation, leading to discrepancies between gene trees and species trees; horizontal gene transfer (HGT), where genes are transferred across different lineages; and introgressive hybridization, where hybrid genes gradually integrate into the parent population, resulting in genetic mixing (16, 19–22). Other factors also influenced species diversification during radiation. For example, adaptive evolution punctuated by positive selection occurs more frequently in radiating lineages than in slowly diversifying ones (23). While ILS, HGT, introgression, and positive selection have been documented in several eukaryotic lineages, such as cichlids (24), wild tomatoes (25), honeybees (26), big cats (27), and *Populus* species (28), their impact on fungal radiation events remains poorly understood.

Here, we characterized the genome-wide patterns and drivers of phylogenetic discordance among the three NTF lineages. Patterns of genome-wide phylogenetic discordance showed that ILS between lineages caused most of the observed discordances. By contrast, introgression and HGT contributed less to the incongruence between species and gene trees. Positive selection of ILS genes associated with growth and trap morphogenesis was also observed. Similar to previous studies of other lineages, our phylogenomic analyses revealed how diverse evolutionary mechanisms contributed to the tempo of NTF evolution and rapid radiation.

## RESULTS

### Extensive phylogenomic discordance among NTF

To investigate the evolutionary history of NTF in Ascomycota, we analyzed 23 NTF genomes and the genome of one non-NTF species as an outgroup (Table S1). The NTF taxa covered three major lineages that underwent radiation and evolved distinct mechanisms of nematode trapping, including 3D adhesive networks (*Arthrobotrys* spp.), 2D adhesive traps (*Dactylellina* spp.), and mechanical traps (*Drechslerella* spp.). *Dactylella cylindrospora*, a member of the Orbiliaceae that does not have trapping capability, was selected as the outgroup.

**Fig 1:**
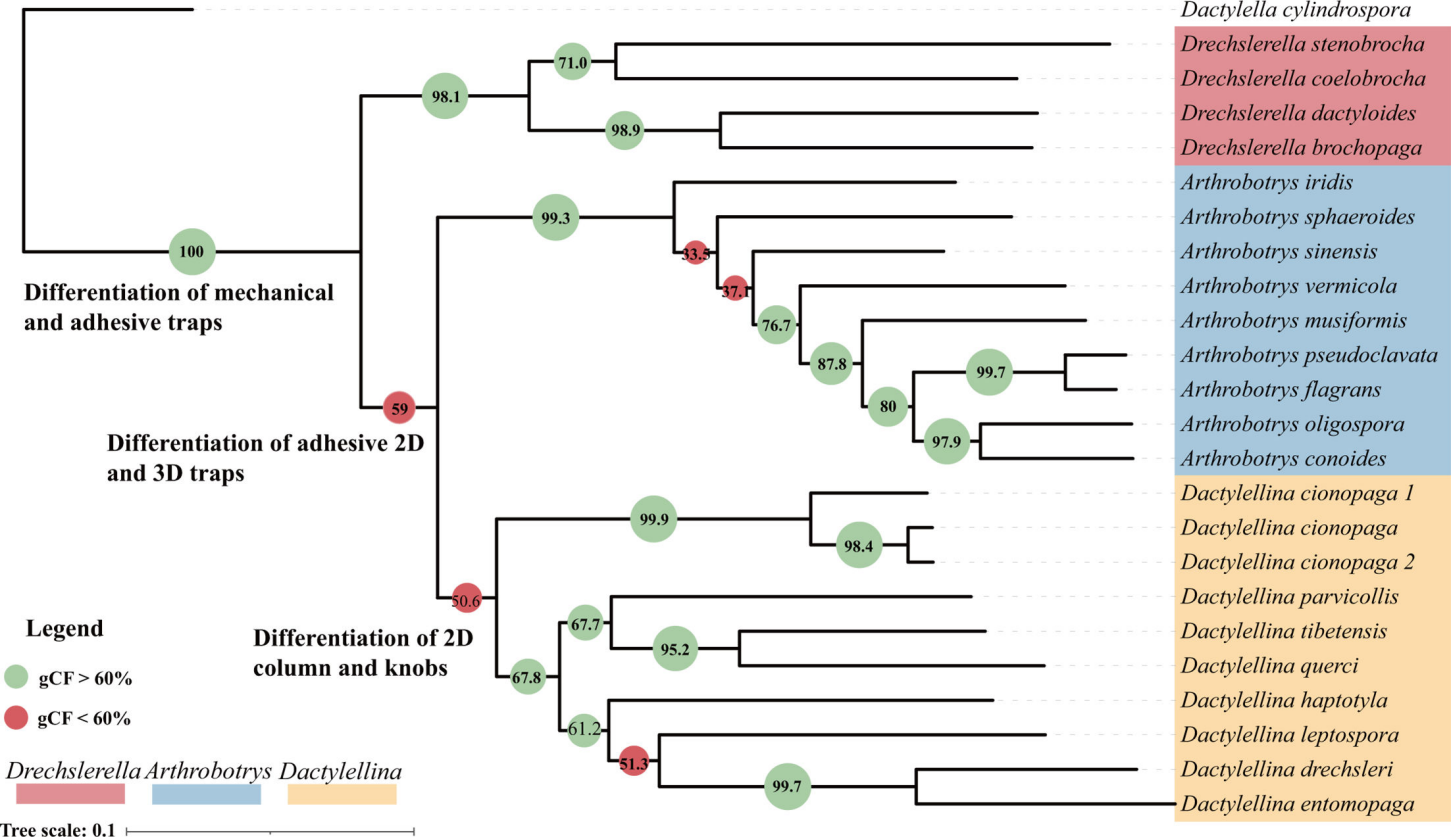
Phylogeny of nematode-trapping fungi. Phylogenic relationships were determined using concatenated nucleotide sequences of the single-copy orthologous genes present in all species. *Dactylella cylindrospora*, a non-NTF species, was used as the outgroup. Bootstrap values were 100% for each node. Gene-concordance factors (gCF) were calculated using IQ-TREE (noted on each node), with green indicating nodes greater than 60% gCF values and red indicating nodes less than 60%.

Single-copy orthologous genes (2,944 in total; Table S2) present in all species were combined to construct a maximum likelihood species tree using two alignment and trimming strategies (Clustal-Omega + ClipKIT and MAFFT + Gblocks) (29–32). The species tree topologies were consistent under both strategies (Fig. 1; Fig. S1), suggesting that our analyses were robust and not significantly affected by analytical errors associated with sequence alignment and trimming. The genome-scale phylogeny was consistent with our previously published multiple-gene phylogeny (33) and strongly supported the placement of *Arthrobotrys* and *Dactylellina* as sister genera (Fig. 1). The species tree supported two notable evolutionary events: the divergence of those forming adhesive traps from the lineage that produces mechanical traps (*Drechslerella*) and the subsequent divergence of 2D (*Dactylellina*) and 3D (*Arthrobotrys*) adhesive traps. The three strains of *Dactylellina cionopaga*, a morphological species with longer branch lengths than other species, suggest that *D. cionopaga* may be a species complex. Further phylogenetic analyses are required to test this hypothesis.

**Fig 2:**
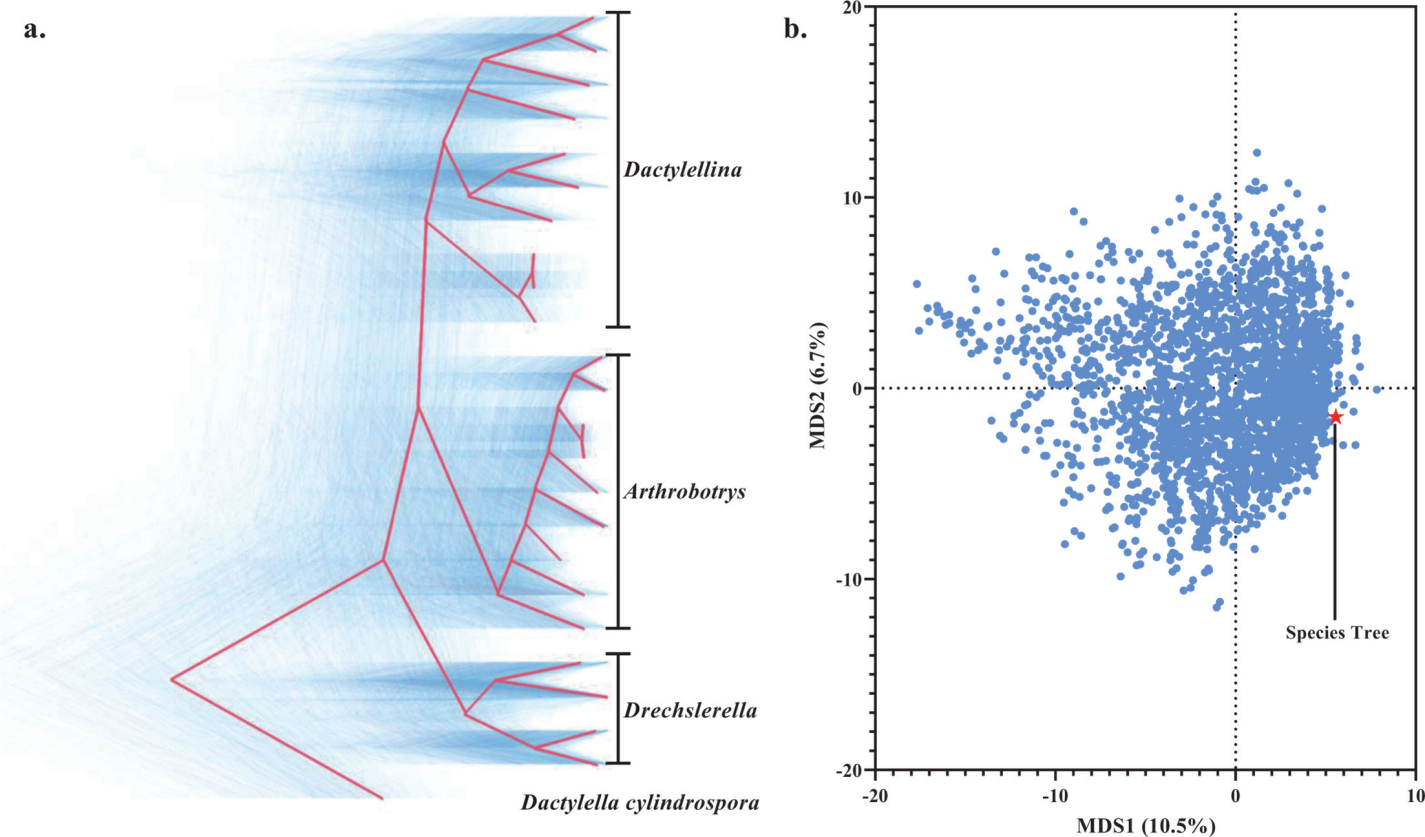
Extensive conflict between the gene trees and the species tree. (a) Densitree plot. Blue represents the gene trees, and red represents the consensus tree inferred by the Densitree software, which is consistent with the topology of the species tree. (b) A plot resulting from MDS analysis illustrates the topological differences between the gene trees (denoted by blue dots) and the species tree (denoted by the red pentagram). The axes (MDS1 and MDS2) represent dimensions in the reduced Euclidean space obtained from the Robinson-Foulds distance matrix calculated between all pairs of gene trees and the species tree. MDS1 and MDS2 have no intrinsic biological significance but are used to visualize the relative differences and conflicts in tree topologies in a low-dimensional space.

Phylogenetic trees of single-copy orthologous genes were also constructed using Clustal-Omega + ClipKIT and MAFFT + Gblocks approaches using a maximum likelihood framework. The resulting trees were largely consistent, suggesting that analytical errors associated with the software choice are minimal (16). Nonetheless, there was abundant discordance between the single-gene trees and the species tree (Fig. 2; Table S2). Densitree plots depicted numerous topological conflicts among the gene trees (24) (Fig. 2a). Robinson-Foulds (RF) distance is a metric that quantifies the differences between two phylogenetic trees. Multidimensional scaling (MDS) analysis based on the RF distance matrix between the species tree and all gene trees revealed extensive phylogenetic conflict (Fig. 2b). Concordance analyses based on IQ-TREE showed a high rate of conflict between gene trees and the species tree at the divergence points between mechanical traps (*Drechslerella*) and adhesive traps, as well as between 2D (*Dactylellina*) and 3D (*Arthrobotrys*) adhesive traps (gene-concordance factors (gCF) <60%, Fig. 1). Two nodes within *Arthrobotrys* and one node within *Dactylellina* had a high conflict (gCF <60%, Fig. 1).

### ILS is largely responsible for phylogenetic discordance

To further rule out analytical sources of error, we identified single gene trees that were consistent between the two alignment and trimming strategies—Clustal-Omega + ClipKIT and MAFFT + Gblocks. Among the 2,944 single-copy orthologous genes, 64 yielded inconsistent gene trees with the two strategies (see Table S2); inconsistent genes, likely subject to analytical errors, were removed from subsequent analyses.

Among the remaining 2,880 gene trees, 488 exhibited bootstrap support below 80% (Table S1), suggesting that errors in phylogenetic inference may have affected these trees. Among the remaining 2,392 trees with high bootstrap support, 978 (40.9%; a group designated as Tree1) supported the species tree, whereas 1,414 (59.1%) were inconsistent with the species tree.

**Fig 3:**
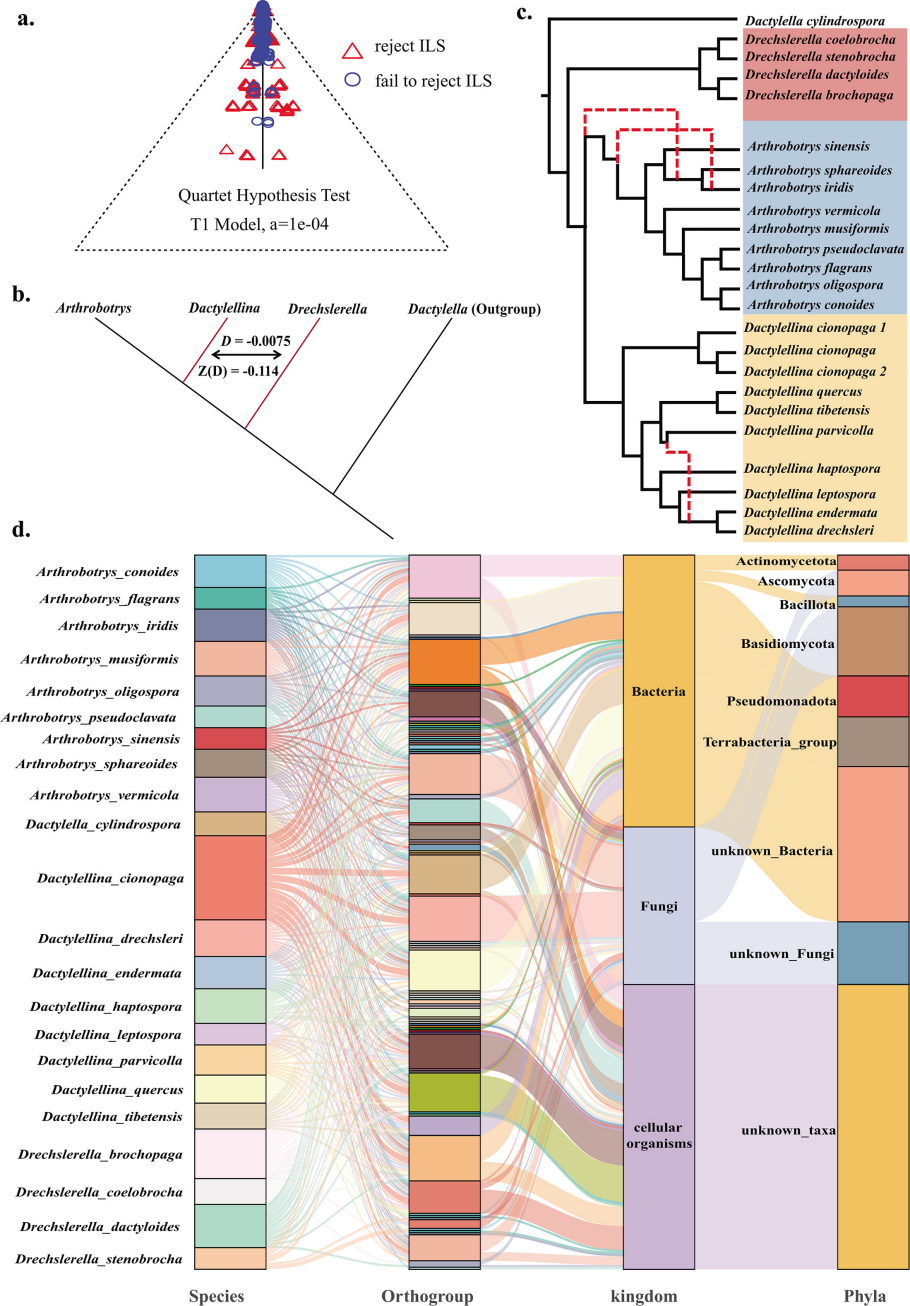
Origins of conflict between the gene trees and the species tree. (a) ILS analysis based on the MSC analysis. Specifically, each vertex represents a particular four-taxa tree topology, and the position of each point within the triangle is determined by the proportions of the three topologies among all gene trees for the four taxa. Blue circles represent four-taxa scenarios in which the topology can be explained solely by the ILS. Red triangles represent scenarios in which this hypothesis is rejected, indicating that the topology is explained by other factors. The closer the blue circles are to the center of the triangle, the stronger the influence of ILS. (b) Schematic representation of *D*-statistic results. The *D*-statistic was derived from the ABBA-BABA test, which detects introgression by comparing the counts of ABBA and BABA SNP patterns across the genome. For the lineages *Arthrobotrys* (P1), *Dactylellina* (P2), *Drechslerella* (P3), and an outgroup (O), ABBA sites are those where P2 and P3 share a derived allele while P1 and the outgroup have the ancestral allele, and BABA sites are where P1 and P3 share the derived allele while P2 and the outgroup have the ancestral allele. The results showed a non-significant *D* value, indicating no significant introgression events between P2 and P3, as well as between P1 and P3. (c) Reticulate phylogenetic tree inferred by Phylonet, with red indicating gene introgression sites. When the number of hybridization events was set to three, the tree inferred by PhyloNet matched the species tree and the fit was optimal. (d) Sankey diagram depicting suspected HGT events among NTF and the predicted sources of genes. The left column represents different NTF species, the middle column represents gene families (orthogroups), and the right column represents the predicted sources of genes, including different kingdoms and phyla. Each flow line indicates a possible path for gene transfer between species, with colors representing different species and gene families.

The Multispecies Coalescent (MSC) model was employed to investigate whether the observed topologies of the gene trees across sets of four taxa could be attributed to ILS (34). Specifically, to ensure each four-taxa combination, the outgroup was fixed, and three taxa were selected from the remaining 23, which resulted in 1,771 unique four-taxa combinations. Using 1,414 gene trees that showed discordance with the species tree, we performed hypothesis testing for each four-taxa combination. The null hypothesis was that the gene tree discordance was caused by ILS, while the alternative hypothesis was that the discordance was due to other factors, such as introgression or horizontal gene transfer (HGT). We compared the observed gene tree distributions with those predicted using the MSC model for each four-taxa combination. At the 0.0001 significance level, if the observed distribution did not significantly deviate from the predicted distribution under the MSC model, we failed to reject the null hypothesis. More specifically, our results showed that 81.3% of the four-taxa scenarios did not reject the null hypothesis, indicating that ILS was the primary factor shaping topology in these cases. Conversely, 18.7% of the scenarios rejected the null hypothesis (Fig. 3a), suggesting that other evolutionary modes, such as introgression and HGT, might have influenced the history of these loci.

Examination of genome-wide *D*-statistics analysis (also known as the ABBA-BABA test) (35, 36), which tests for introgression, revealed insignificant amounts of introgression among the three NTF lineages (Fig. 3b; *D* = −0.0075, Z = −0.114). However, phylogenetic network analysis revealed two introgression events within the *Arthrobotrys* lineage and one within the *Dactylellina* lineage. Each event potentially involved one or more genes (Fig. 3c). Notably, these event nodes showed high degrees of conflict between the gene and species trees, which may partly stem from introgression (Fig. 1).

Among the 1,414 genes displaying topological structures that conflict with the species tree, 36 appeared to have been acquired *via* HGT. These HGT genes predominantly originated from bacteria, with Pseudomonadota being the main donor phylum. Some HGT events from fungi, particularly from the sister phylum Basidiomycota, were also observed (Fig. 3d).

**Fig 4:**
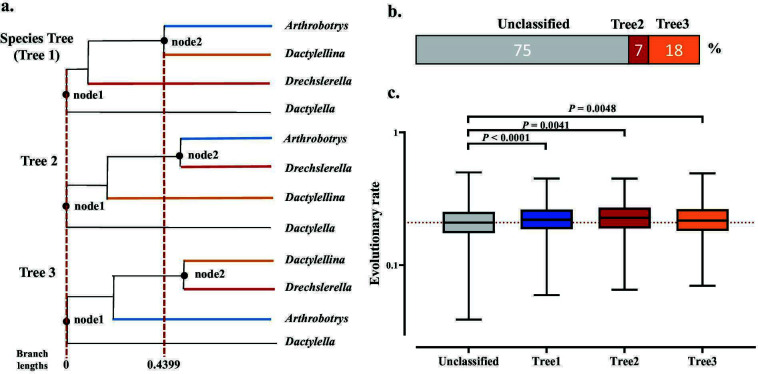
Divergence nodes and cumulative branch lengths for the three NTF genera. (a) Schematic representation of the topological structures of the three gene trees and their divergence branch lengths. The root node was labeled as node 1, and the second divergence node of the three lineages was labeled as node 2. The dashed red line represents the branch length scale of the divergence nodes in the species tree. Each evolutionary branch is colored to correspond with its genus name. The branch lengths shown are the average branch lengths of the gene trees supporting each topological structure, with statistical tests performed on the three groups of data. (b) Stacked bar chart showing the proportions of the three types of gene tree topologies inconsistent with the species tree. (c) Box plot of cumulative branch lengths for the four types of gene trees.

The remaining 1,378 trees were categorized into three groups (Fig. 4a): 7.0% (97) placed *Arthrobotrys* and *Drechslerella* as sister groups (Tree2); 18.0% (245) clustered *Drechslerella* and *Dactylellina* (Tree3); and 75.0% (1,036) did not align with their corresponding generic clades (designated as Unclassified).

The branch lengths at the divergence nodes of the gene trees likely affected by ILS were longer than those in the species tree, a significant signal supporting ILS (37). We compared the divergent branch lengths between the ancestral node (node1) and the next divergence node (node2), which represents the duration of nematode-trapping device divergence in the three different types of gene trees (Fig. 4a). The mean divergent branch lengths for Tree2 and Tree3 (0.5746 and 0.5895, respectively) were significantly shorter than that for the species tree (0.4399, *P* < 0.0001), supporting the contribution of ILS to the divergence of the three NTF lineages.

The phylogenetic conflicts in those categorized as “Unclassified” (1,036 trees) were likely caused by ILS (Fig. 4b). The MSC analysis indicated that 84.44% of the conflicts in the four taxa could not reject the hypothesis that they arose from ILS (Fig. S2). ILS events involve random fixation of ancestral sequences, leading to many topologies spanning the NTF lineages. A substantial number of gene trees exhibiting inconsistency with the lineages may be due to the stochastic nature of ILS. At the same time, the lack of correspondence between these gene trees and the species tree suggests that these are more ancient ILS events. Compared to Tree1, Tree2, and Tree3, the Unclassified-type trees have significantly shorter cumulative branch lengths (Fig. 4c), suggesting lower evolutionary rates (38) (Table S3).

### ILS genes under positive selection are broadly associated with growth and trap morphogenesis

**Fig 5:**
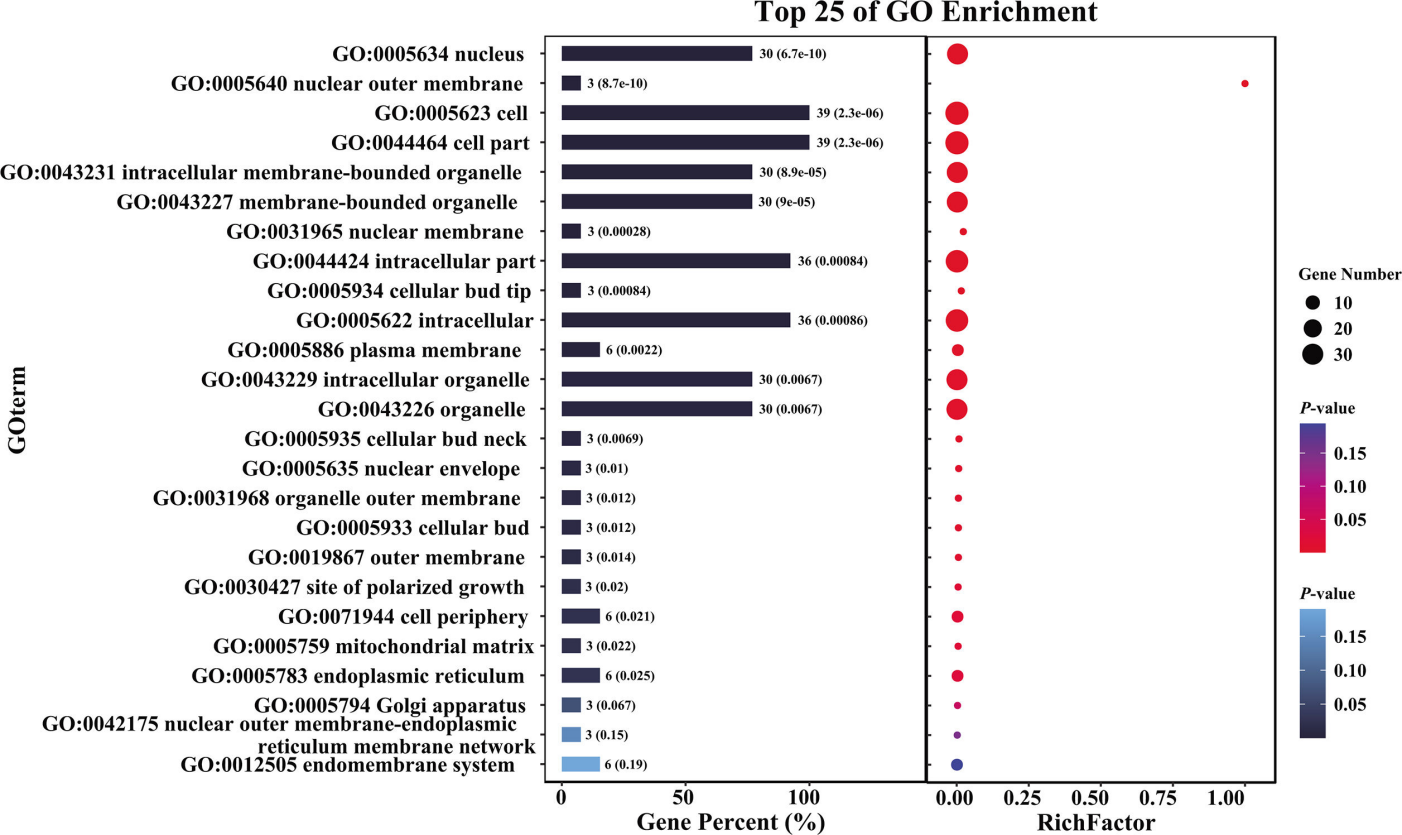
Functional enrichment analysis. Functional enrichment analysis of ILS genes that are linked to the divergence of the three NTF lineages and display signs of significant positive selection. The Gene Ontology (GO) terms enriched among those associated with the cell membrane system and polarity division are shown. The bar chart on the left shows GO terms, with the length of each bar representing the percentage of genes associated with that term. The numbers on the bars indicate the gene count and the *P*-value for each term. The dot plot on the right shows the enrichment analysis results, with the x-axis representing the RichFactor (the ratio of the number of genes in the term to the total number of annotated genes). The size of the dots corresponds to the number of genes, while the color indicates the significance (*P*-value) of the enrichment, with red indicating higher significance and blue indicating lower significance.

Natural selection during rapid evolutionary radiation frequently leads to accelerated gene evolution and resulting phenotypic changes (23, 39). Positive selection among ILS genes was detected using CodeML with the site model. Sixteen single-copy orthologous genes exhibited signs of significant positive selection (Table S2) and were enriched for functions related to the cell membrane system and cellular polar division (Fig. 5; Tables S3 and S4). For example, functions annotated to the *Saccharomyces cerevisiae* HMX1 (YLR205C) gene (Tables S3 and S4) are associated with the cell membrane system, including the plasma membrane (GO:0005886, annotated with the PHO81 gene, an ankyrin repeat protein nuc-2), nuclear outer membrane (GO:0005640), outer membrane (GO:0019867), and endoplasmic reticulum (GO:0005783). Functions related to cellular polarity division, including the cellular bud tip (GO:0005934), neck (GO:0005935), cellular bud (GO:0005933), and site of polarized growth (GO:0030427), were annotated with the PH domain-containing protein gene (Tables S3 and S4).

Among the conserved genes categorized as “Unclassified,” 35 families showed significant evidence of positive selection (Table S2). The functions of these gene families are primarily enriched in processes related to RNA polymerase, cell nucleus, and transcription (Fig. S3; Table S5), such as those encoding DNA polymerase, DNA ligase, RNA exonuclease, DnaJ domain proteins, and transcription factors (Table S6). These functions are crucial for conserved cytological processes.

## DISCUSSION

We investigated the evolutionary history of carnivorous NTF in Ascomycota by analyzing the genome-wide pattern of phylogenetic discordance and positive selection using the genomes of 21 species (23 strains) representing three NTF lineages. We generated the first genome-scale species tree of these NTF. The genome-scale species tree (Fig. 1) was consistent with the previously published phylogenetic trees (9, 33); however, we found extensive phylogenetic discordance across the genome and nodes of the species tree. The ILS between lineages caused 81.3% of the phylogenetic discordance, while 18.7% was attributed to post-speciation introgression within the lineage or HGT. The reticulate phylogenetic inference indicates that introgression only led to differentiation within certain NTF genera. Although HGT events caused conflict between gene trees and the species tree, they were not the primary drivers of widespread phylogenetic inconsistencies. These results suggest that the PT extinction led to rapid stochastic fixation of ancestral polymorphisms and diverged along the NTF lineages. Subsequent positive selection accelerated the evolution of genes associated with carnivory. Several HGT events may have contributed to genetic polymorphism in carnivores. Moreover, gene flow between NTF lineages was restricted, with limited introgression within each lineage.

The main sources of phylogenetic discordance between the gene and species trees are ILS, introgression, and HGT. Genome-wide signatures of ILS and introgression can be distinguished because the coalescence times for regions under ILS should be older than the speciation events. By contrast, hybridization is post-speciation event (19). The observation that the branch lengths from the ancestral nodes to the lineage differentiation nodes in Tree2 and Tree3 are longer than those in the species tree supports the hypothesis that ILS is the primary cause of the observed phylogenetic discordance. ILS causes ancestral genetic polymorphisms to persist during rapid speciation (40), and ILS events have been detected in many lineages, including marsupials (19), peat moss (41), butterflies (42), filamentous fungi (18, 43), and eared seals (20). Our study indicates that the evolution of NTF represents a new case of ILS-driven evolution.

We also observed signals of introgression within *Arthrobotrys* and *Dactylellina* with the occurrence of a reticulate phylogenetic relationship within each lineage. Consequently, the effect of introgression was more pronounced among the closely related species within the generic lineage. The role of gene introgression events in species evolution has garnered increasing attention because numerous studies have highlighted their significant effects on ecological adaptability and evolution in species such as primates, butterflies (42), gray snub-nosed monkeys (44), and foxes (45). Future studies should explore the effects of introgression within each NTF lineage.

Some inconsistencies between the gene and species trees were caused by HGT. Most HGT genes originated from bacteria, but some originated from fungi in the phylum Basidiomycota. Although HGT events may not be the main factor driving the divergence of NTF lineages, they typically introduce traits that play a crucial role in evolution (46), which may also hold true for carnivorous fungi. Functional characterization of such genes should be performed to assess their significance in the evolution of NTF (13).

The most conflict-rich regions tend to be associated with the highest rates of phenotypic innovation, which have been detected in six clades of vertebrates and plants (47). The most conflict-rich nodes in this study also coincide with the differentiation nodes of NTF nematode traps, which also implies that these genes undergoing ILS may be associated with morphological innovation in NTF, although additional studies are needed. We found that some ILS genes, especially those involved in the cell membrane system are involved in trap morphogenesis (48, 49) and inflation of the constricting ring (50), underwent positive selection. The role of positive selection in the adaptive radiation of cichlids, wild tomatoes, and Jaltomata has also been demonstrated, although there are gene tree discordances in their evolutionary processes (24, 25, 51). Using gene trees for each ILS gene instead of the species tree in our positive selection analysis helped reduce the risk of false positives. This underscores the significance of positive selection as an evolutionary driver that accelerates the adaptive radiation of carnivorous fungi. Additional analysis to strengthen the association between ILS and positive selection among NTF will help shed light on the strength of these processes shaping NTF biology.

Gene tree discordance represents another source of substitution rate variation that can lead to false inferences regarding positive selection (52). Genes linked to adaptive traits might not align with the species tree, causing changes in substitution rates and potentially misleading conclusions about positive selection. Therefore, the interpretation of positive selection and adaptive radiation requires caution. Our study detected positive selection in the genes associated with carnivorous traits. The use of gene trees for each ILS gene instead of the species tree in our positive selection analysis helped to reduce the risk of false positives. This underscores the significance of positive selection as an evolutionary driver that accelerates the adaptive radiation of carnivorous fungi.

Many genes that did not align with their corresponding generic clades are likely to have originated prior to the divergence of the three NTF lineages. ILS typically results in the random retention of ancestral sequences (53, 54), and this stochastic process is responsible for the generation of gene trees that do not align with the clades of the lineage. The significantly shorter cumulative branch lengths observed in these gene trees (Fig. 4c) suggest their ancient origin and conservation, indicating their role in conserved functions related to basic life processes rather than those associated with carnivorous lifestyles. Our findings highlight the importance of these genes.

### Conclusion

The evolutionary history of NTF in Ascomycota, a phylum to which most known carnivorous fungi belong, was investigated through phylogenomic analyses. Their evolution was facilitated by the PT extinction, which led to rapid radiation driven by ILS, coupled with positive selection of the genes associated with various carnivorous traits between generic lineages, and introgression within each lineage of two genera that form adhesive traps. These analyses advanced our understanding of the genetic mechanisms underlying fungal adaptive radiation and evolution.

## MATERIALS AND METHODS

### Genome mining

Genomes with the published protein-coding gene predictions were obtained from the National Center for Biotechnology Information (Table S1, https://www.ncbi.nlm.nih.gov/bioproject/791178). Considering the frequent expansion of gene families during fungal evolution, only single-copy genes present in all species were used in this study. In total, 2,944 gene groups were identified (Tables S2 and S3) using OrthoFinder v 2.5.6 (55). The nucleotide and protein sequences of these genes were then matched. Conserved protein domains were predicted using pfam-scan (56). Gene Ontology (GO) terms based on the functional domains were obtained using pfam2go (http://geneontology.org/external2go/pfam2go). The detailed gene functions were predicted using InterProScan (http://www.ebi.ac.uk/interpro/).

### Phylogenetic analyses

To minimize the impact of phylogenetic inference errors on subsequent analyses, we employed two methodologies for phylogenetic analysis, resulting in two sets of species and gene trees.

The first approach involved aligning the nucleotide sequences of all single-copy orthologous genes using MAFFT v 7.520 and Gblock v 0.91b (29, 30). The combined sequences were used to construct species trees using IQ-TREE v 2.2.2.7 with 1,000 replicates (57). Individual gene trees based on nucleotide sequences were constructed using IQ-TREE v 2.2.2.7 with 1,000 replicates. The species and gene trees were rooted using the corresponding sequences of *D. cylindrospora*.

The second approach involved aligning the nucleotide sequences of all single-copy orthologous genes using Clustal-Omega v 1.2.4 and ClipKIT v 2.2.2 (31, 32). The combined sequences were used to construct species trees using IQ-TREE v 2.2.2.6 with 1,000 replicates (57). Individual gene trees based on nucleotide sequences were constructed using IQ-TREE with 1,000 replicates. The species and gene trees were rooted using the corresponding sequences of *D. cylindrospora*.

We also conducted parallel analyses using amino acid sequences. Although the results were consistent, the phylogenetic trees based on amino acid sequences had lower bootstrap support values (Fig. S4; Table S1). Thus, we chose nucleotide sequence-based trees, as they provided more robust and reliable support for our analyses. We conducted the same analysis using amino acid sequences.

The tree types were identified using classify_tree.py, a tool available on GitHub (https://github.com/dengweihx/classifytree). By comparing the classification results of the two datasets, only gene trees that were consistent across both data sets were used for further analysis.

The incongruence coefficients of gene trees at each branch node of the species tree were calculated using IQ-TREE v 2.2.2.6, and the species tree was presented using Interactive Tree Of Life (iTOL) v5 (58). Densitree plots of conflicting gene tree topologies were drawn using DensiTree v 3.0.2 (59) (https://www.cs.auckland.ac.nz/~remco/DensiTree/download.html). Pairwise Robinson-Foulds (RF) distances between gene trees were calculated using the ape package for R 4.1.3, and the RF distances were then analyzed and plotted by MDS (34, 60). The evolutionary rate of each gene tree was calculated using PhyKIT (38).

### Incomplete lineage sorting analysis

ILS signals were detected by calculating the branch lengths of the differentiated nodes of the gene trees using the Internal Branch Statistics feature of the PhyKIT toolkit (38). Differences in branch length were determined using the *t*-test. *D* values were detected by the z-test against whole-genome backgrounds (35, 36) (see https://github.com/simonhmartin/ tutorials/tree/master/ABBA_BABA_whole_genome for *D* statistics). ILS analyses based on the four-taxon branch length chi-square test were performed and plotted using the MSCquartets package R 4.1.3 (61). Reticulated phylogenetic inference based on the InferNetwork_MP model was performed using PhyloNet v 3.8.2 (https://phylogenomics.rice.edu/html/tutorials.html). Detection of genome-wide HGT events was performed using HGTector2 (https://github.com/qiyunlab/HGTector).

### Analysis of positive selection

Positive selection on 2,944 single-copy orthologous genes was evaluated using CodeML and PAML (62) based on the GWideCodeML package for Python 3.10.12 (https://github.com/lauguma/gwidecodeml). The dn/ds values for each clade were calculated using the site model. To correct for errors in substitution rate estimation due to ILS, we performed branch site model calculations for the genes subjected to ILS based on their gene trees. Results from the GO term enrichment analysis were presented using the clusterProfiler package for R 4.3.2 (60).

## Data Availability

All the genomic data analyzed in this study are available in GenBank. Specifically, we sequenced 18 genomes, and the remaining 6 genomes were downloaded from GenBank. The accession numbers of the genomes are listed in Table S1.

## SUPPLEMENTAL MATERIAL

Supplemental Figures mbio.02133-24-s0001.docx Figures S1 to S4.

Supplemental Tables mbio.02133-24-s0002.xlsx Tables S1 to S7.

ASM does not own the copyrights to Supplemental Material that may be linked to, or accessed through, an article. The authors have granted ASM a non-exclusive, world-wide license to publish the Supplemental Material files. Please contact the corresponding author directly for reuse.
