# Supplementary material for: Characterization of genome-wide phylogenetic conflict uncovers evolutionary modes of carnivorous fungi
Source: mBio. 2024 Aug 29;15(10):e02133-24. doi: 10.1128/mbio.02133-24 (PMC11481490; doi:10.1128/mbio.02133-24)
Supplement: Supplemental Figures — Figures S1 to S4. [file mbio.02133-24-s0001.docx]

Supporting information

**Characterization of Genome-wide Phylogenetic Conflict Uncovers Evolutionary Modes of Carnivorous Fungi**

Weiwei Zhang^1,2,3,#,^ Yani Fan^2,3,#^, Wei Deng^1,#^, Yue Chen^1^, Shunxian Wang^1^, Seogchan Kang^4^, Jacob Lucas Steenwyk^5^, Meichun Xiang^2,3^*, Xingzhong Liu^1,2,^*

^1^State Key Laboratory of Medicinal Chemical Biology, Key Laboratory of Molecular Microbiology and Technology, Department of Microbiology, College of Life Science, Nankai University, Tianjin 300071, China

^2^State Key Laboratory of Mycology, Institute of Microbiology, Chinese Academy of Sciences, Beijing 100101, China

^3^University of Chinese Academy of Sciences, Beijing 100049, China

^4^Department of Plant Pathology & Environmental Microbiology, The Pennsylvania State University, PA 16802, USA

^5^ Howards Hughes Medical Institute and Department of Molecular and Cell Biology, University of California, Berkeley, CA 94720, USA

^#^These authors contributed equally

*Correspondence authors: Meichun Xiang (xiangmc@im.ac.cn) and Xingzhong Liu (liuxz@nankai.edu.cn).

**
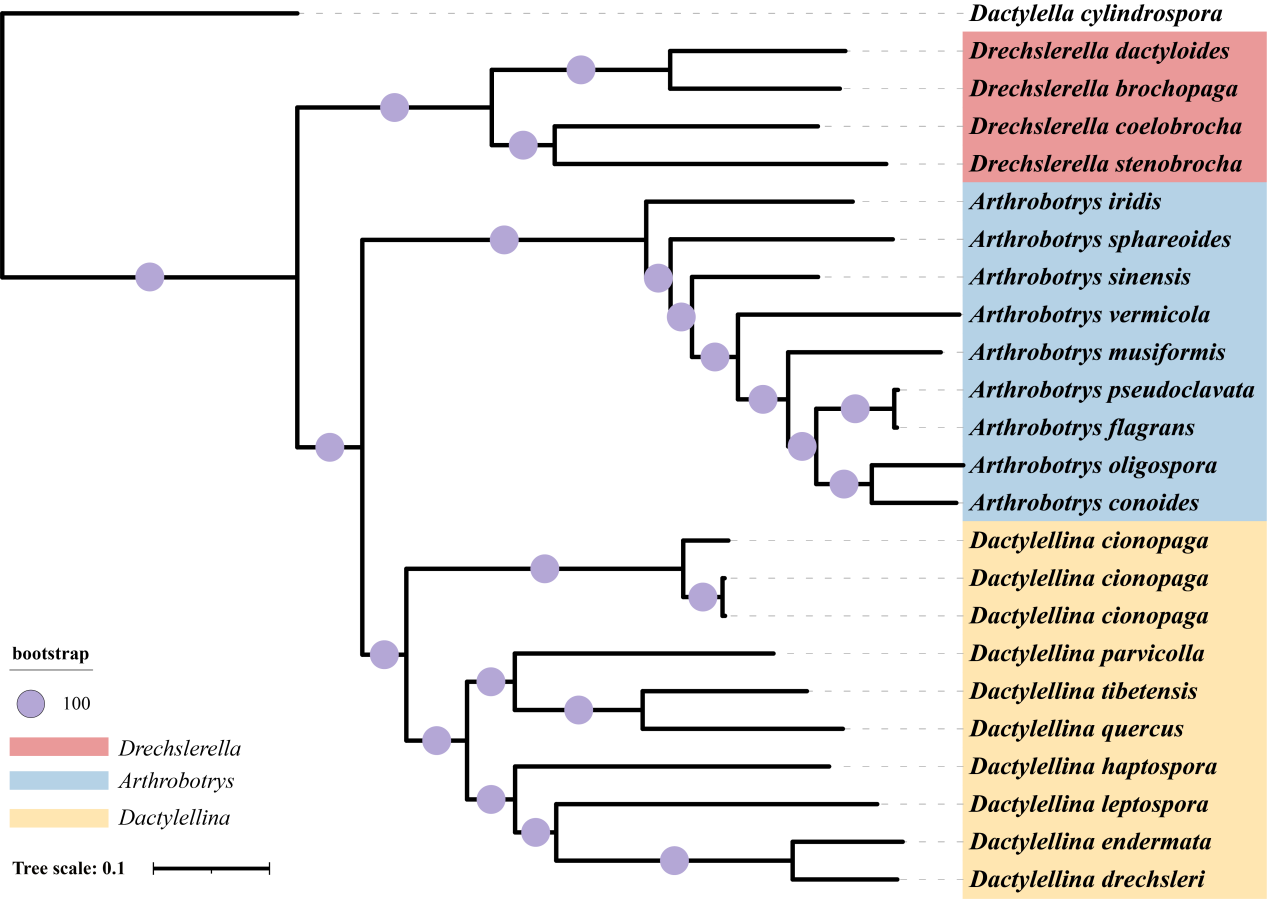
**

**Figure S1. Species tree2 of nematode-trapping fungi.** Their phylogenetic relationships were determined using concatenated nucleotide sequences of the single-copy orthologous genes, aligned with MAFFT, trimmed with Gblock, and a maximum likelihood tree was constructed using IQ-TREE. Bootstrap values were 100% on each node. *Dactylella cylindrospora*, a non-NTF species, was used as the outgroup.

**
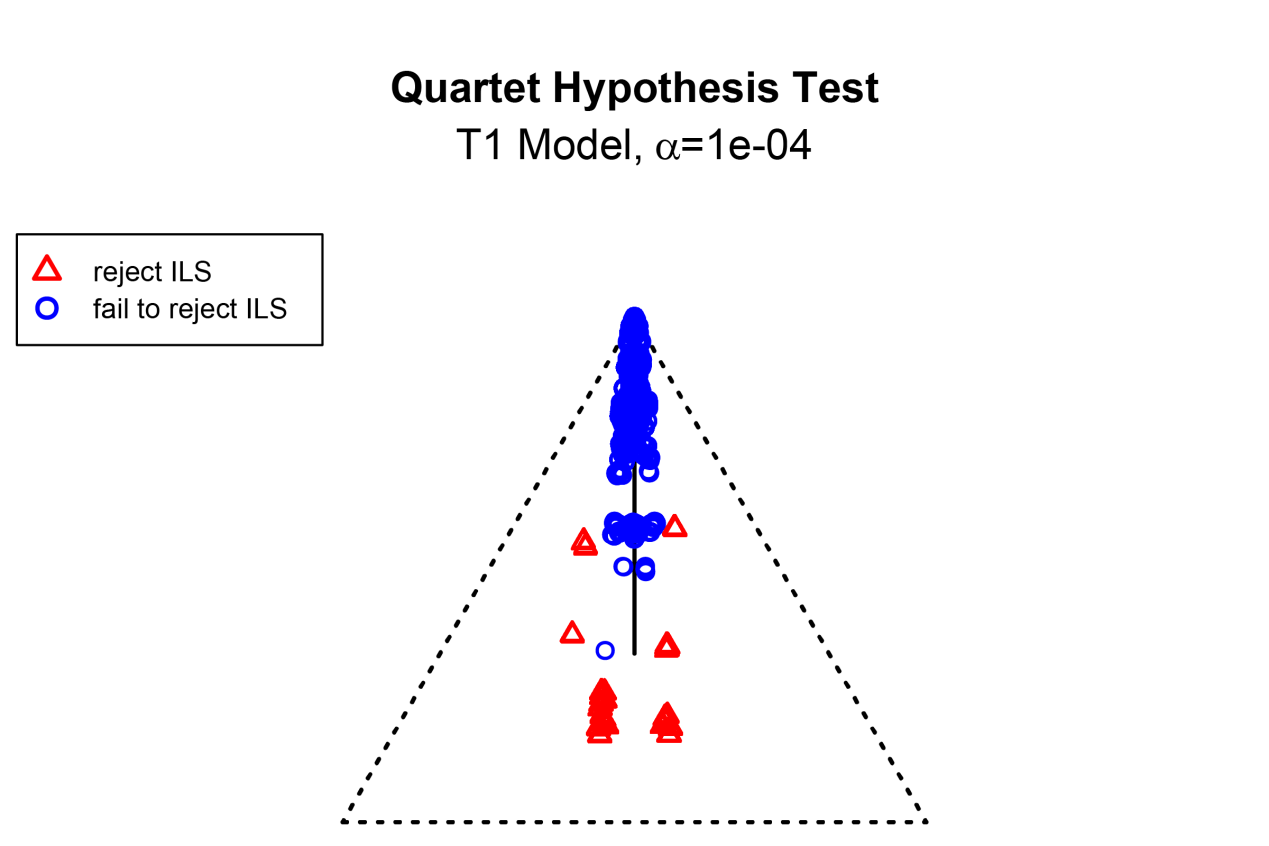
****Figure S2.** **Multispecies coalescent (MSC) analysis of "Unclassified" type gene trees to evaluate the involvement of ILS.** Blue circles represent four-taxa scenarios in which the topology can be explained solely by the ILS. Red triangles represent scenarios in which this hypothesis is rejected, indicating that the topology is explained by other factors. The closer the blue circles to the center of the triangle, the stronger the influence of ILS.


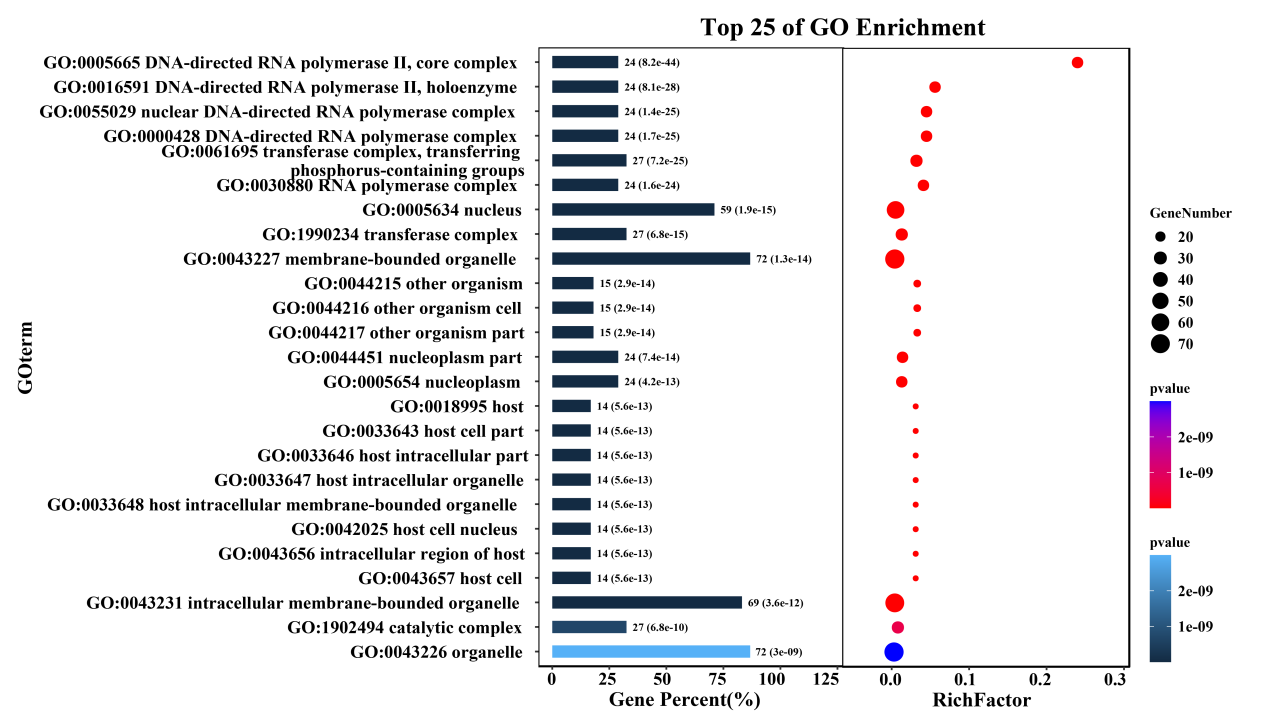


**Figure S3.** **GO Functional enrichment analysis of the genes that belong to "Unclassified" Type trees.**

**
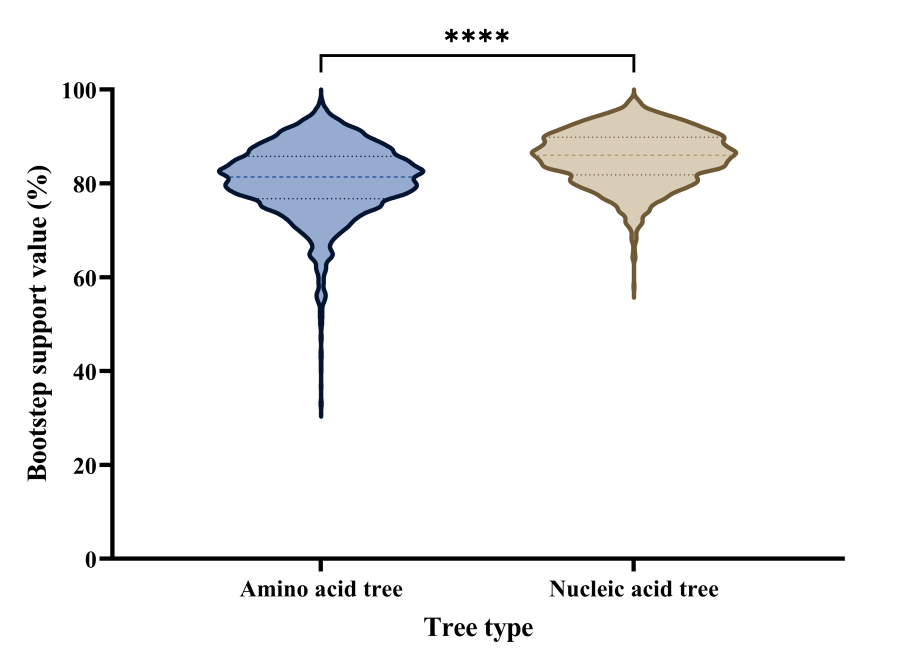
Figure S4. Differences in Bootstrap Support between Nucleic Acid Trees and Amino Acid Trees.** The average bootstrap support for 2944 amino acid trees is 81.38%, with a range of 32.85% to 97.76% and a standard deviation of 7.66. For 2944 nucleic acid trees, the average bootstrap support is 85.56%, with a range of 57.81% to 98.43% and a standard deviation of 5.71. A paired sample t-test was conducted, showing that the average bootstrap support for nucleic acid trees is significantly higher than that for amino acid trees (t = 48.86, df = 2943, P < 0.0001).
